# R-Propranolol Has Broad-Spectrum Anti-Coronavirus Activity and Suppresses Factors Involved in Pathogenic Angiogenesis

**DOI:** 10.3390/ijms24054588

**Published:** 2023-02-27

**Authors:** Melissa Thaler, Clarisse Salgado-Benvindo, Anouk Leijs, Ali Tas, Dennis K. Ninaber, Jack L. Arbiser, Eric J. Snijder, Martijn J. van Hemert

**Affiliations:** 1 Department of Medical Microbiology, Leiden University Medical Center, 2333 ZA Leiden, The Netherlands; 2 Department of Pulmonology, Leiden University Medical Center, 2333 ZA Leiden, The Netherlands; 3 Department of Dermatology, Emory University School of Medicine, Atlanta, GA 30322, USA; 4 Division of Dermatology, Veterans Affairs Medical Center, Decatur, GA 30033, USA

**Keywords:** propranolol, R-propranolol, antiviral, coronavirus, SARS-CoV-2, angiogenesis, *angptl4*

## Abstract

The SARS-CoV-2 pandemic highlighted the need for broad-spectrum antivirals to increase our preparedness. Patients often require treatment by the time that blocking virus replication is less effective. Therefore, therapy should not only aim to inhibit the virus, but also to suppress pathogenic host responses, e.g., leading to microvascular changes and pulmonary damage. Clinical studies have previously linked SARS-CoV-2 infection to pathogenic intussusceptive angiogenesis in the lungs, involving the upregulation of angiogenic factors such as ANGPTL4. The β-blocker propranolol is used to suppress aberrant ANGPTL4 expression in the treatment of hemangiomas. Therefore, we investigated the effect of propranolol on SARS-CoV-2 infection and the expression of ANGPTL4. SARS-CoV-2 upregulated ANGPTL4 in endothelial and other cells, which could be suppressed with R-propranolol. The compound also inhibited the replication of SARS-CoV-2 in Vero-E6 cells and reduced the viral load by up to ~2 logs in various cell lines and primary human airway epithelial cultures. R-propranolol was as effective as S-propranolol but lacks the latter’s undesired β-blocker activity. R-propranolol also inhibited SARS-CoV and MERS-CoV. It inhibited a post-entry step of the replication cycle, likely via host factors. The broad-spectrum antiviral effect and suppression of factors involved in pathogenic angiogenesis make R-propranolol an interesting molecule to further explore for the treatment of coronavirus infections.

## 1. Introduction

Severe acute respiratory syndrome coronavirus 2 (SARS-CoV-2) emerged in 2019 and expanded the class of highly pathogenic human coronaviruses, which also includes SARS-CoV and Middle East respiratory syndrome-CoV (MERS-CoV). Although the lethality of SARS-CoV-2 infection is lower compared to SARS-CoV and MERS-CoV, its transmissibility is much higher and its fast spread led to an unprecedented pandemic and an enormous burden on healthcare systems worldwide. The gravity of its associated disease, COVID-19, has called for the fast development of antiviral therapies or the repurposing of existing approved drugs. Although patients often present with only mild or even no symptoms, elderly people or those with underlying conditions can experience severe pulmonary damage and acute respiratory distress syndrome (ARDS) that can include endothelial and vascular damage, thromboinflammation, neutrophilic and macrophage dysfunction, immunopathology, and intussusceptive angiogenesis [1]. The epithelium–endothelium environment during infection and the pathological changes in the lung microvascular system have been investigated in several studies [2,3,4]. Early in the pandemic, morphological changes such as intussusceptive angiogenesis in the lungs of deceased patients were described as distinct features of COVID-19 compared to influenza virus-infected or healthy lungs [5]. Although the formation of new blood vessels is usually a well-regulated process, for example, in wound healing, unregulated angiogenesis can have negative implications, leading to pathogenesis [4]. Intussusceptive angiogenesis is a rapid process of blood vessel neoformation that happens due to the splitting of an existing vessel into two, as opposed to sprouting angiogenesis. Although the implications of vascular changes for response and repair to different lung pathologies are not well understood, it has been reported that viral infections can dysregulate these processes by creating a pro-angiogenic environment of inflammation and hypoxia [6,7]. In line with these microvascular changes, transcriptional analysis identified the upregulation of angiogenic factors, such as *angptl4* and *VEGFA*, to a magnitude that appears unique to COVID-19 [5,8]. Angiogenic dysregulation and therapeutic interventions to control this process have been widely studied in the field of cancer research. One common type of tumor involving dysregulated blood vessel formation is infantile hemangioma. Propranolol, an approved and widely used non-selective β-blocker, is used to treat this condition, as this compound has also been shown to inhibit pro-angiogenic (transcription) factors [9,10]. Propranolol is a mixture of two enantiomers, R- and S-propranolol. As opposed to its S- counterpart, R-propranolol does not exert beta-blocker activity but was shown to reduce the angiogenic factor ANGPTL4 (more efficiently) in hemangioma stem cells [9]. Anti-angiogenic drugs are also used as a safe treatment for idiopathic pulmonary fibrosis [11]. Bevacizumab, a monoclonal antibody with anti-angiogenic properties, was also evaluated in a clinical trial for its efficacy in reducing angiogenesis and vascular damage in COVID-19 patients [12]. Therefore, in this study, we aimed to investigate the effect of R-propranolol on the expression of angiogenesis factors that are induced by SARS-CoV-2 infection and study its effect on virus replication. Besides an effect on ANGPTL4 expression, we, surprisingly, also uncovered a potent antiviral effect of propranolol against a broad spectrum of highly pathogenic coronaviruses.

The emergence of SARS-CoV-2 as the third highly pathogenic coronavirus within two decades shows the importance of developing broad-spectrum antivirals to prepare us for future outbreaks. The repurposing of already approved and safe drugs can provide a faster and more efficient way into clinics when an outbreak calls for a swift response. Our study suggests that the approved drug R-propranolol could function as a ‘double-edged sword’, inhibiting both virus replication and pathogenic host responses to infection. It might, therefore, be an interesting drug to further explore in clinical trials.

## 2. Results

### 2.1. SARS-CoV-2 Infection Upregulates Angiogenic Factor angptl4 in Endothelial Cells

**Figure 1:**
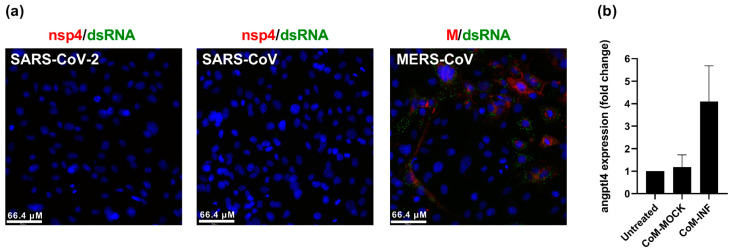
Infection of Hulec-5a lung endothelial cells with various coronaviruses and expression of angiogenic factor angptl4. (**a**) Hulec-5a cells were infected with SARS-CoV-2, SARS-CoV, and MERS-CoV (multiplicity of infection (MOI) 5), and after 48 h, the cells were fixed and stained with rabbit polyclonal anti-SARS-CoV nsp4 protein antibody for SARS-CoV-2 and SARS-CoV, rabbit polyclonal anti-MERS-CoV M protein antibody for MERS-CoV, mouse monoclonal anti-dsRNA antibody (J2), and Hoechst, and visualized by immunofluorescence microscopy. Images are representative of results from 3 independent experiments. (**b**) Hulec-5a cells were incubated with conditioned medium from uninfected (CoM-MOCK) or infected (CoM-INF) Calu-3 lung epithelial cells for 24 h (diluted 1:2 with endothelial cell medium). Intracellular RNA was isolated to quantify *angptl4* expression by RT-qPCR, using PGK1 as a reference gene. Changes in *angptl4* expression were normalized to untreated Hulec-5a cells (Untreated). The mean ± SEM of 3 independent experiments is shown.

To investigate whether the lung pathology-associated upregulation of angiogenic factors that was reported in COVID-19 patients could also be observed in cell culture, we used Hulec-5a human lung endothelial cells as a relevant model for microvascular changes [13]. We monitored the expression of *angptl4*, which encodes the Angiopoietin-like 4 protein and was reported to be upregulated in the lungs of COVID-19 patients [5]. Hulec-5a cells could not be infected with SARS-CoV-2, which has been previously reported [2]. These cells were also not susceptible to SARS-CoV but could be infected with MERS-CoV, as indicated by the positive immunofluorescence staining for the viral membrane protein (M) and dsRNA (Figure 1a). Since Hulec-5a cells could not be infected with SARS-CoV-2, we used conditioned medium from SARS-CoV-2-infected Calu-3 lung epithelial cells to mimic the epithelium–endothelium environment during infection. When the Hulec-5a cells were incubated with conditioned medium from mock-infected Calu-3 cells, *angptl4* expression remained unchanged compared to untreated Hulec-5a cells (Figure 1b). When Hulec-5a cells were incubated with conditioned medium from infected Calu-3 cells, we measured a fourfold increase in *angptl4* expression by RT-qPCR, in line with the reported effect of infection on the transcriptional changes in endothelial cells of patients.

### 2.2. R-Propranolol Downregulates Expression of the Angiogenic Factor angptl4

**Figure 2:**
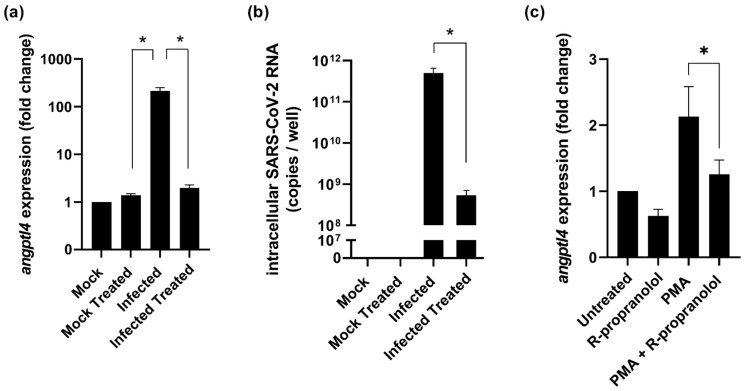
Effect of R-propranolol on the upregulation of *angptl4* by infection or PMA treatment. Vero E6 cells were infected with SARS-CoV-2 (MOI 1) in the absence or presence of 50 µM R-propranolol. (**a**) The fold increases in *angptl4* RNA levels compared to untreated uninfected cells (Mock) and (**b**) intracellular viral RNA copies were quantified by RT-qPCR at 16 hpi. *n* = 2 independent experiments. (**c**) Hulec-5a cells were treated with 0.1 µM PMA; 6 h later, 50 µM R-propranolol was added; and 12 h later, the increase in *anptl4* expression was quantified by RT-qPCR. *n* = 2 independent experiments. For normalization, the levels of the reference gene PGK1 were measured by RT-qPCR in all experiments. Mean ± SEM is shown. Statistical analysis was conducted using a ratio-paired *t*-test and significant differences are indicated by * *p* < 0.05.

Angiogenic factors such as ANGPTL4 are also upregulated in infantile hemangiomas and their expression can be suppressed with the drug propranolol, or more specifically, the stereoisomer R-propranolol. R-propranolol exerts its inhibitory effect on angiogenic factors without having beta-blocker activity [9] and, therefore, we investigated the effect of R-propranolol on the expression of SARS-CoV-2-induced angiogenesis factors. Since Hulec-5a cells were not susceptible to SARS-CoV-2 (Figure 1), we tested if *angptl4* expression was also upregulated during infection of Vero E6 cells and whether treatment with R-propranolol would suppress it. Vero E6 cells were treated with 50 µM of R-propranolol and were infected with SARS-CoV-2 at an MOI of 1. Analysis of total intracellular RNA isolated from infected cells at 16 hpi showed an over 100-fold increase in *angptl4* expression (Figure 2a). When the infected cells were treated with R-propranolol, *angptl4* expression was not increased and was very similar to that in the uninfected cells. Interestingly, when we measured the intracellular viral RNA copies in those samples, we observed a 100-fold reduction in the viral load in R-propranolol-treated cells compared to the infected untreated cells (Figure 2b). To uncouple the direct effect of R-propranolol on *angptl4* expression from its effect on virus replication, we used the chemical inducer phorbol-12-myristate-13-acetate (PMA) to increase *angptl4* expression. In Hulec-5a cells, we observed that R-propranolol caused a moderate but significant downregulation of the PMA-induced increase in *angptl4* expression (Figure 2c). This suggested that at least part of the effect of R-propranolol on this angiogenic factor is independent of its effect on virus replication.

### 2.3. R- and S-Propranolol Inhibit SARS-CoV-2 Replication in Cell Culture

**Figure 3:**
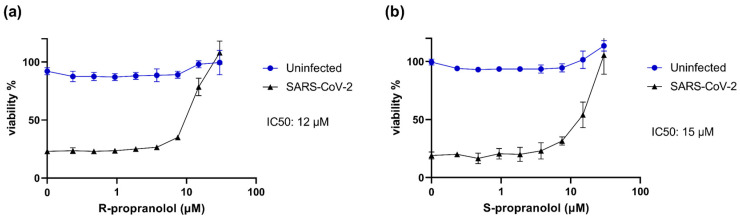
Effect of R-propranolol and S-propranolol on SARS-CoV-2-induced cytopathic effect in Vero E6 cells. CPE reduction assays were carried out on Vero E6 cells infected with SARS-CoV-2 (MOI of 0.015). Cells were pretreated with and infected in the presence of (**a**) R-propranolol, or (**b**) S-propranolol. At 72 hpi, cell viability was determined by MTS assay. *n* = 2 independent experiments. Cell viability of uninfected compound-treated cells was measured in parallel to assess the cytotoxicity of the compound. Mean ± SEM is shown. The 50% inhibitory concentration (IC50) was determined by non-linear regression with GraphPad Prism 6.

The quantification of intracellular viral RNA in the experiments to study *angptl4* expression suggested that R-propranolol inhibited virus replication. Therefore, we assessed the effect of the compound in more detail in various antiviral assays. The marketed drug propranolol contains a mixture of the stereoisomers S- and R-propranolol. Therefore, we assessed the effect of both S- and R-propranolol in cytopathic effect (CPE) reduction assays with SARS-CoV-2. Vero E6 cells were infected at a low MOI in the presence of the compound, and after 72 h, cell viability was measured by MTS assay. R-propranolol protected the cells from infection with an IC50 of 12 µM (Figure 3a). S-propranolol inhibited SARS-CoV-2 replication with an IC50 of 15 µM (Figure 3b). We observed no cytotoxicity in the uninfected cells that were treated in parallel with the same increasing concentrations of compounds. Since R-propranolol was slightly more effective than S-propranolol and is devoid of the undesired beta-blocker activity, we decided to perform all further experiments with R-propranolol.

### 2.4. R-Propranolol Inhibits SARS-CoV-2 Replication in Various Cell Lines and Air–Liquid Interface-Cultured Primary Human Airway Epithelial Cells

**Figure 4:**
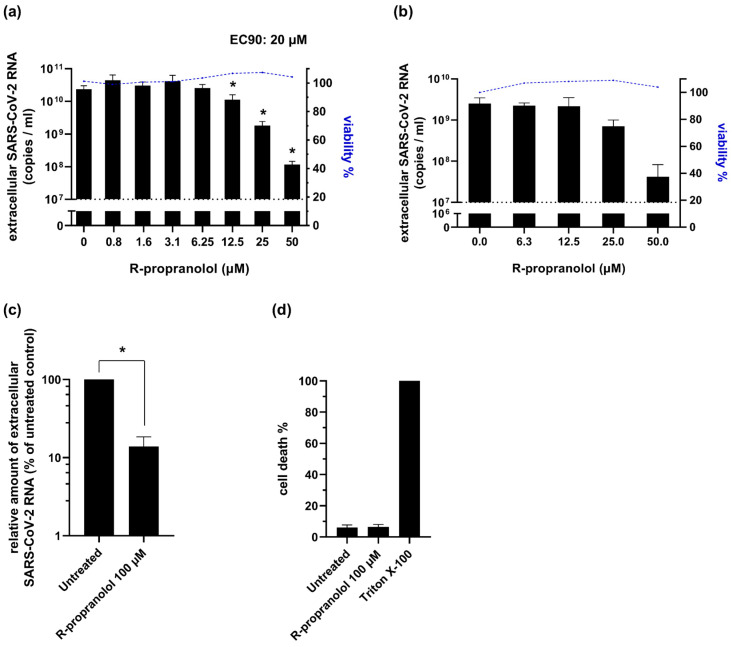
Effect of R-propranolol on replication of SARS-CoV-2 in Vero E6 and H1299-hACE2 cells and primary human bronchial epithelial cell cultures grown at the air–liquid interface (ALI-PBEC). (**a**) Viral load reduction assay on Vero E6 cells. Cells were infected with an MOI of 1 and at 16 hpi, extracellular viral RNA copy numbers were determined by RT-qPCR. *n* = 3 independent experiments. (**b**) H1299-hACE2 cells were infected at MOI 0.001 and the viral load in the medium at 48 hpi was determined by RT-qPCR. *n* = 2 independent experiments. In parallel, the cell viability of uninfected Vero E6 and H1299-hACE2 cells treated with R-propranolol was measured by MTS assay and data were normalized to the untreated uninfected cells (% viability). (**c**) ALI-PBEC were treated with 100 µM R-propranolol and inoculated with SARS-CoV-2 for 2 h (MOI ~0.1) in the presence of the compound. After removal of the inoculum, the viral load in the apical wash was determined at 48 hpi. R-propranolol remained present in the basal medium until 48 hpi. *n* = 3 independent experiments. Mean ± SEM is shown for all experiments. (**d**) In ALI-PBEC, cytotoxicity was monitored by quantifying the release of LDH and data were normalized to ALI-PBEC treated with Triton X-100 (100% cell death). *n* = 2 independent experiments. Statistical analysis was conducted using two-way ANOVA with a Tukey/Bonferroni post hoc test or in (**c**) with a *t*-test. Significant differences are indicated by * *p* < 0.05.

To validate the antiviral effect of R-propranolol that was observed in the CPE reduction assays (Figure 3), we performed viral load reduction assays. Vero E6 cells were pretreated with the compound, infected at an MOI of 1, and at 16 hpi the viral load was determined by quantifying the number of extracellular viral RNA copies by RT-qPCR. In this single replication cycle experiment, R-propranolol caused a significant and up to a 100-fold reduction in extracellular viral RNA copies (Figure 4a), with a 90% effective concentration (EC90) of 20 µM. To assess whether the compound was also able to prevent the spread of infection in human lung cells, we infected the human lung epithelial cell line H1299-hACE2 at an MOI of 0.001, treated it with the compound, and measured the viral load after multiple rounds of replication at 48 hpi (Figure 4b). In this infection model, we also observed a 100-fold reduction in the viral load and an EC90 of 26 µM. Measurement of the cell viability by MTS assay showed that the compound caused no cytotoxicity at the concentrations used in both cell lines.

To analyze the efficacy of R-propranolol in a more advanced infection model, we used primary bronchial epithelial cells that were cultured at the air–liquid interface (ALI-PBEC). This infection model efficiently recapitulates the pseudostratified epithelium of the lung and its infection by coronaviruses (Thaler et al., manuscript under revision). ALI-PBEC were infected with SARS-CoV-2 (MOI of ~0.1) and treated with 100 µM of R-propranolol on the apical side of the cells during the 2 h inoculation with the virus. Infected control cells were treated with PBS instead of the compound (“untreated” control). The compound was also added to the medium on the basal side of the cells and remained present until 48 hpi when the samples were harvested. We observed a significant reduction in the viral load for the R-propranolol-treated cells compared to the untreated controls (Figure 4c). Measurement of cell death (by LDH release) in the untreated and treated ALI-PBEC indicated that R-propranolol caused no significant cytotoxicity under these conditions (Figure 4d).

### 2.5. R-Propranolol Inhibits SARS-CoV-2 at a Post-Entry Step of the Replication Cycle

**Figure 5:**
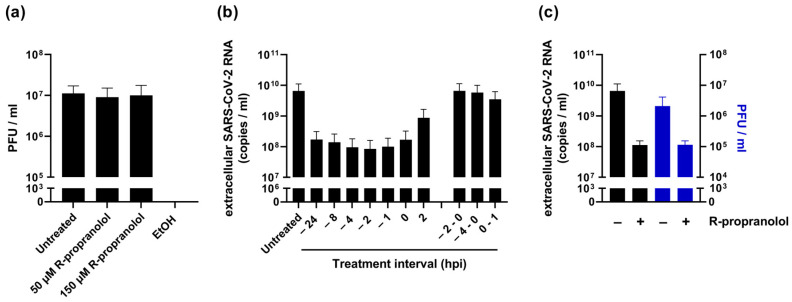
R-propranolol inhibits the replication of SARS-CoV-2 at a post-entry step. (**a**) Effect of the compound on infectivity of SARS-CoV-2. Virus was incubated with either 50 or 150 µM R-propranolol, 70% ethanol, or medium (untreated control) for 1 h at 37 °C. Remaining infectivity was measured by plaque assay. *n* = 3 independent experiments. (**b**) Time-of-addition assay with Vero E6 cells infected at MOI 1 and R-propranolol treatments during the time intervals indicated on the *x*-axis or initiated at the indicated time points, all relative to the time of infection (t = 0). Compound remained present until 16 hpi when supernatant was harvested for quantification of extracellular viral RNA by RT-qPCR. *n* = 2 independent experiments. (**c**) Supernatant of infected Vero E6 cells at 16 hpi, untreated or treated with 50 µM R-propranolol, was analyzed by RT-qPCR and plaque assay. Mean ± SEM of 3 independent experiments is shown.

To elucidate the mode of action of R-propranolol, we first evaluated whether the compound affected the infectivity of viral particles, i.e., whether it exhibited virucidal activity. Therefore, we incubated SARS-CoV-2 with 50 or 150 µM of the compound for 1 h at 37 °C before assessing the (remaining) number of infectious particles by plaque assay. Control treatment with 70% ethanol led to the complete inactivation of the virus, but R-propranolol had no effect on the infectious titer compared to the untreated virus stock (Figure 5a).

To elucidate which step of the viral life cycle was affected by R-propranolol, we performed a time-of-addition assay (Figure 5b). The strongest reduction in the viral load was observed when the cells were pretreated and the compound remained present during infection until the time of harvesting. Although less effective, treatment initiated at 2 hpi still reduced the viral load, suggesting that the compound did not target the attachment/entry of the virus. This was further supported by the observation that pretreatment alone (2 or 4 h prior to infection until the time of infection) or the presence of the compound in the inoculum only during infection (0–1 hpi) did not reduce the viral load. Together, these results suggested that R-propranolol inhibited a post-entry step of the replication cycle, possibly via its effect on a host factor(s).

To obtain more insights into the mode of action, we compared the specific infectivity of the treated and untreated samples (Figure 5c). Infectious progeny and extracellular genome copies were reduced to the same extent by R-propranolol treatment, suggesting that the compound did not affect the infectivity of viral genomes (i.e., it did not compromise genome integrity) and did not affect the infectivity of released particles. It led to an overall reduction in the release of infectious particles.

### 2.6. R-Propranolol Has Broad-Spectrum Antiviral Activity against Different Coronaviruses

**Figure 6:**
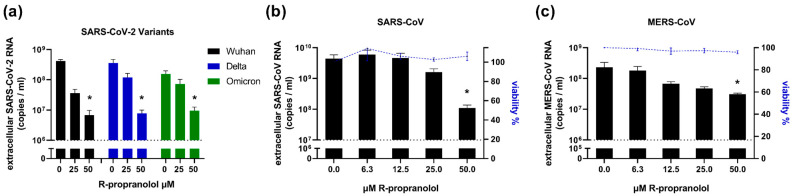
Effect of R-propranolol on replication of SARS-CoV-2 variants, SARS-CoV, and MERS-CoV. (**a**) Viral load reduction assay on Vero E6 cells with SARS-CoV-2 variants. The viral load at 16 hpi was determined by RT-qPCR and cell viability was monitored in parallel. *n* = 2 independent experiments. (**b**) Viral load reduction assay with SARS-CoV in Vero E6 cells infected at MOI 1. Viral load at 16 hpi was determined by RT-qPCR and cell viability was monitored in parallel. *n* = 3 independent experiments. (**c**) HuH-7 cells were infected with MERS-CoV (MOI 1) and at 16 hpi, viral load was determined by RT-qPCR. *n* = 3 independent experiments. Mean ± SEM is shown. Statistical analysis was conducted using two-way ANOVA with a Tukey/Bonferroni post hoc test. Significant differences are indicated by * *p* < 0.05. Cell viability of uninfected cells treated with the compound was determined in parallel using MTS assay and data were normalized to untreated uninfected cells (100% viability).

We assessed the activity of R-propranolol against various SARS-CoV-2 variants in viral load reduction assays and found that the Delta and Omicron variants were also inhibited by R-propranolol (Figure 6a). We then evaluated its effect on different highly pathogenic coronaviruses. The compound also inhibited SARS-CoV replication in Vero E6 cells (Figure 6b) and MERS-CoV replication in HuH-7 cells (Figure 6c), suggesting it has a broad spectrum of activity against coronaviruses.

## 3. Discussion

Despite the unprecedented successes of vaccine development in response to the SARS-CoV-2 pandemic, the demand for safe and effective antiviral drugs remains. Not only to treat COVID-19 patients but also to better prepare us for future coronavirus outbreaks, as such drugs could be used prophylactically, post-exposure, or in outbreak settings when vaccines for new threats are not yet available. Since COVID-19 patients often present with serious symptoms rather late, there is a need not only for drugs that directly inhibit virus replication but also for therapeutics that target the (pathogenic) host responses to infection. The hallmarks of severe SARS-CoV-2 infection are pulmonary inflammation, tissue damage, and microvascular changes in the lung. Pathogenic angiogenesis and upregulation of pro-angiogenic factors such as ANGPTL4 have been observed in patients with severe SARS-CoV-2 or, to a lesser extent, influenza virus infections [5]. Recently, increased ANGPTL4 plasma levels were associated with higher proportions of ARDS and increased mortality [8]. In mice deficient in ANGPTL4, influenza virus infection led to less pulmonary damage [14], suggesting that suppression of the SARS-CoV-2-induced upregulation of ANGPTL4 may be a strategy to counteract the pathogenic angiogenesis that can be caused by SARS-CoV-2 infection. In this study, we explored the potential therapeutic effect of R-propranolol on COVID-19 pathophysiology and uncovered its broad-spectrum antiviral effect against coronaviruses.

The approved drug propranolol is a non-selective β-adrenergic blocker that is mainly used to treat cardiovascular problems. However, it also has a beta-blocker-independent effect on angiogenesis and is therefore used to treat infantile hemangiomas [9]. Propranolol consists of the stereoisomers R-propranolol and S-propranolol of which R-propranolol is devoid of beta-blocker activity. R-propranolol was shown to reduce the pro-angiogenic factor ANGPTL4 in a murine hemangioma model, without having beta-blocker activity and thus circumventing the potential side effects that could be caused by the beta-blocker activity of S-propranolol. Therefore, we focused our study on R-propranolol and confirmed the inhibitory effect of this compound on chemically induced *angptl4* expression in lung endothelial cells. Lung endothelial cells were not susceptible to SARS-CoV-2, but the treatment of endothelial cells with conditioned medium from SARS-CoV-2-infected epithelial cells caused an increase in *angptl4* expression. This underlines the impact of the epithelium–endothelium crosstalk during infection and the consequences for the microvascular system. *Angptl4* expression was also upregulated in SARS-CoV-2-infected Vero E6 cells and this effect could be suppressed by treatment with R-propranolol. Future studies will need to address the effect of infection and R-propranolol in more advanced (organoid and in vivo) models to assess whether the compound could have a therapeutic effect on exacerbated angiogenesis.

Besides the effect on the expression of virus-induced angiogenic factors, we also observed that R-propranolol inhibits virus replication. Our study uncovered for the first time a direct antiviral effect of R-propranolol that supports previous clinical observations of the apparent (indirect) beneficial effect of propranolol on the outcome of viral infections [15]. R-propranolol treatment of infected Vero E6 cells protected them from cytopathic effects with an EC50 of 12 µM, which was slightly more effective than S-propranolol, which had an EC50 of 15 µM. Treatment of infected cells also led to a significant reduction in extracellular viral RNA copies with an EC90 of 20 µM and the compound also reduced the viral load by 2 logs in a lung cell line infection model. Finally, we employed primary bronchial epithelial cells cultured at the air–liquid interface as the most relevant infection system, as it efficiently reconstitutes the pseudostratified lung epithelium and its infection. In this model, R-propranolol also reduced the viral load in the apical wash of the culture without causing any noticeable cytotoxicity.

Furthermore, we could show that R-propranolol has broad-spectrum activity against different SARS-CoV-2 variants, including Delta and Omicron, and it also inhibited other highly pathogenic coronaviruses, i.e., SARS-CoV and MERS-CoV. The compound had no virucidal effect and time-of-addition assays suggested that it inhibited a post-entry step of the replication cycle.

Its broad-spectrum activity and its enhanced activity upon pretreatment suggest that R-propranolol likely inhibits viral replication via one or more host factors. These likely play no role in the attachment or entry of the viral particle since there was no reduction in the viral load when the compound was present only during infection (in the inoculum). R-propranolol reduced the amount of infectious progeny that was released from cells but did not appear to affect the specific infectivity, suggesting it does not affect the infectivity of particles or integrity of the genome (e.g., it does not lead to mutations or defects in capping). While we were preparing this manuscript, a preprint was published on the effect of R-propranolol on SARS-CoV-2 and MHV replication, including the observation of an inhibitory effect on MHV replication in a mouse model [16]. The findings of this study were in line with our data and it was suggested that the compound affected replication complex formation through an effect on phospholipid synthesis, which corroborates our results (in time-of-addition assays). Previous studies on propranolol-induced changes in gene expression in endothelial cells also included genes involved in lipid and sterol metabolism and ubiquitination [17]. Propranolol has also been shown to affect various other host factors and signaling pathways, including inhibition of the RAS/RAF/ERK and AKT pathways [18,19]. The inhibition of factors involved in these signaling pathways was also shown to affect SARS-CoV-2 replication [20]. Therefore, to unravel the compound’s mode of action, more in-depth research is required to elucidate which host factor(s) or pathway(s) contribute to the R-propranolol-mediated inhibition of coronavirus replication.

It is also important to keep in mind that most of the studies on the activity of propranolol or its separate stereoisomers have been conducted in the context of cancer research. Therefore, these findings might not be directly translatable to non-cancer models and need to be investigated in proper infection models. The widespread use of propranolol renders it a safe and interesting drug for further investigations, and the use of the enantiomer R-propranolol, which has no beta-blocker activity, lowers the risk of possible negative side effects. Its inhibition of virus replication and suppression of detrimental host responses to infection make R-propranolol an interesting compound for further evaluation in clinical studies.

## 4. Materials and Methods

### 4.1. Compounds and Cell Culture

R-(+)-Propranolol hydrochloride, S-(+)-Propranolol hydrochloride, and phorbol-12-myristate-13-acetate (PMA) were purchased from MedChemExpress and dissolved in DMSO.

Vero E6 cells and HuH-7 cells were cultured as previously described [21]. Human lung cell line H1299-hACE2 is described elsewhere (Salgado-Benvindo et al., manuscript in preparation). These cells were cultured in Dulbecco’s modified Eagle’s medium with 4.5 g/L glucose with L-glutamin (DMEM; Lonza, Basel, Switzerland) supplemented with 10% fetal calf serum (FCS) (CapriCorn Scientific, Ebsdorfergrund, Germany), 100 U/mL of Penicillin/Streptomycin (P/S) (Sigma-Aldrich, St. Louis, MO, USA), and 1200 µg/mL G418 for selection (InvivoGen, San Diego, CA, USA). Calu-3 cells (ATCC, HTB-55TM) were cultured in Eagle’s minimum essential medium (EMEM, Lonza) supplemented with 9% FCS, 1% non-essential amino acids (NEAA, Sigma-Aldrich), 2 mM L-glutamine (Sigma-Aldrich), 1 mM sodium pyruvate (Sigma-Aldrich), and 100 U/mL of P/S.

Infections of Vero E6 cells, HuH-7 cells, H1299-hACE2 cells, and Calu-3 cells were performed in Eagle’s minimal essential medium with 25 mM HEPES (EMEM; Lonza) supplemented with 2% FCS, 2 mM L-glutamine (Sigma-Aldrich), and 100 U/mL of P/S (referred to as infection medium).

Primary human bronchial epithelial cells (PBEC) were isolated and cultured as previously described [22]. Hulec-5a cells were purchased from ATCC and cultured and infected in EGM^TM^-2 MV Microvascular Endothelial Cell Growth Medium-2 BulletKit^TM^ (Lonza). Hulec-5a cells were used between Passages 2 and 6. Infections of Hulec-5a cells were carried out in a culture medium and treatment with PMA was carried out in an infection medium.

All cell cultures were maintained at 37 °C in an atmosphere of 5% CO_2_.

### 4.2. Virus Stocks

All experiments with infectious SARS-CoV, SARS-CoV-2, or MERS-CoV were performed at the LUMC biosafety level 3 facilities. For Vero E6 infections, the clinical isolate SARS-CoV-2/Leiden-0002 (isolated at LUMC during the first wave of the COVID-19 pandemic in March 2020 (GenBank: MT510999.1)) was used. For H1299-hACE2 and ALI-PBEC infections, SARS-CoV-2/Leiden-0008 (isolated at LUMC during the first wave of the COVID-19 pandemic in March 2020 (GenBank: MT705206.1)) was used, as it was not adapted to Vero E6 cells with regard to the spike S1/S2 cleavage site (confirmed by NGS sequencing). SARS-CoV-2 variant BA.1 (Omicron) was obtained from RIVM (strain hCoV-19/Netherlands/NH-RIVM-72291/2021, lineage B.1.1.529), and variant B.1.617 (Delta) was obtained from the University of Leuven. SARS-CoV-2/Leiden-0002 (Passage 3), SARS-CoV-2/Leiden-0008 (Passage 2), SARS-CoV isolate Frankfurt 1 [23] (Passage 4), Omicron (Passage 3), and Delta (Passage 4) variants were grown in Vero E6 cells. MERS-CoV (N3/Jordan) (GenBank: KJ614529.1) (Passage 3) was grown on HuH-7 cells. Virus titers were determined by plaque assay on Vero E6 cells, and for MERS-CoV, on HuH-7 cells, as described previously [24].

### 4.3. Endothelial Cell Infection

Hulec-5a cells were seeded on glass coverslips at a density of 8 × 10^4^ cells/well in a 12-well plate and infected with SARS-CoV, SARS-CoV-2, or MERS-CoV at an MOI of 5 in 500 µL medium per well. After 48 h, cells were fixed and processed as previously described [25]. Cells were stained with rabbit polyclonal anti-SARS-CoV nsp4 protein antibody (FGQ4) for SARS-CoV-2 and SARS-CoV, rabbit polyclonal anti-MERS-CoV M protein antibody (R9004) for MERS-CoV, and with mouse monoclonal anti-dsRNA antibody (J2). Secondary antibodies used were a Cy3-conjugated donkey anti-rabbit IgG antibody (Jackson ImmunoResearch Laboratories, West Grove, PA, USA) and an Alexa488-conjugated goat anti-mouse IgG antibody (Invitrogen, Waltham, MA, USA).

### 4.4. Endothelial Cell (Drug) Treatments

Hulec-5a cells were incubated with conditioned medium from infected Calu-3 lung epithelial cells (CoM-INF) for 24 h (diluted 1:2 with fresh endothelial cell medium). As a control, we used conditioned medium from uninfected Calu-3 cells (CoM-MOCK). Intracellular RNA was isolated to quantify *angptl4* expression by RT-qPCR. To produce CoM-INF, Calu-3 cells were seeded at a density of 2.4 × 10^5^ cells/well in 6-well plates and infected with SARS-CoV-2 (Leiden-008) at an MOI of 1 for 2 h at 37 °C on a rocking platform. To prepare CoM-MOCK, Calu-3 cells were incubated with medium (instead of virus) for the same amount of time. The inoculum was removed, cells were washed with PBS three times, and 500 µL of infection medium was added to both infected and mock-infected cells. At 48 hpi, medium was harvested and stored at −80 °C.

For treatment with PMA, Hulec-5a cells were seeded at a density of 7 × 10^3^ cells/well in a 96-well plate. After 24 h, the medium was changed to an infection medium. After 16 h, cells were treated with 100 nM PMA for 6 h. Then, 50 µM R-propranolol was added and cells were incubated for 12 h. Control wells were treated with only PMA, R-propranolol, or medium with the DMSO vehicle as control (untreated). Then, intracellular RNA was harvested and *angptl4* expression was quantified by RT-qPCR.

### 4.5. Cytopathic Effect (CPE) Reduction Assay

CPE reduction assay was performed as previously described [26]. Briefly, Vero E6 cells were seeded in 96-well plates at a density of 5 × 10^3^ cells per well. The next day, cells were incubated with 2-fold serial dilutions of R- or S-propranolol starting at 50 µM and subsequently infected. At 3 days post-infection, the CellTiter 96 aqueous nonradioactive cell proliferation kit (Promega, Madison, WI, USA) was used to measure the cell viability of infected (protection) and non-infected cells (assessment of cytotoxicity).

### 4.6. Viral Load Reduction Assays

Viral load reduction assays were performed as previously described [26]. Briefly, Vero E6 cells were infected with SARS-CoV-2, SARS-CoV-2 variants, or SARS-CoV, and HuH-7 cells were infected with MERS-CoV at an MOI of 1. Pretreatment with R-propranolol was performed for 4 h. The DMSO concentration that was present in samples with the highest compound concentration was used as the vehicle control, which is depicted in the graphs as a value of 0 on the x-axis. Alternatively, Vero E6 cells were seeded at a density of 7 × 10^4^ cells/well in 24-well plates (Figure 2a,b). Cells were pretreated for 2 h with 50 µM R-propranolol and infected with SARS-CoV-2 at an MOI of 1. At 16 hpi, total intracellular RNA was harvested and viral RNA copies (intracellular and extracellular) and *angptl4* expression were quantified by multiplex RT-qPCR using PGK1 as the reference gene.

### 4.7. Time-of-Addition Assay

Vero E6 cells were seeded in 24-well cell culture plates at a density of 2 × 10^4^ cells/well. Cells were incubated with 50 µM of R-propranolol in 500 µL infection medium at the indicated timepoints. 48 h after seeding, cells were infected with 8 × 10^4^ PFU of virus per well (MOI 1) in 200 µL of medium. Supernatant was harvested at 16 hpi and extracellular viral RNA copies were measured by RT-qPCR [24].

### 4.8. Virucidal Effect Assay

Vero E6 cells were seeded in 6-well cell culture plates at a density of 3.5 × 10^5^ cells/well. The next day, the virus (3 × 10^5^ PFU) was incubated in a total of 60 µL of only medium, medium with 50 or 150 µM compound, or ethanol (66% end concentration) at 37 °C. After incubation for 1 h, 50 µL samples were serially diluted to lower the compound and ethanol concentration to a level that did not inhibit the subsequent plaque assay to determine the remaining infectious virus titer [24].

### 4.9. Viral Infection of ALI-PBEC

The apical side of the cells was washed with 200 µL warm PBS for 10 min at 37 °C to remove excess mucus and cellular debris and the basal medium was refreshed prior to infection. Cells were infected with 100,000 PFU of SARS-CoV-2 in 200 µL PBS (with compound or DMSO as control) per insert for 2 h at 37 °C on a rocking platform (estimated multiplicity of infection (MOI) of 0.1). Compound or DMSO as vehicle control was also present in the basal medium until the time of harvesting. For noninfected (mock) controls, the same procedure was performed with only PBS. After removal of the inoculum, the apical side of the cells was washed three times with PBS. At 48 hpi, 200 µL of PBS was added to the apical side of the cells and after incubation for 10 min at 37 °C, supernatant was harvested to quantify the viral load by RT-qPCR.

### 4.10. RNA Isolation and RT-qPCR

RNA was isolated either using Tripure isolation reagent (Sigma-Aldrich) or by magnetic bead isolation. Briefly, 20 µL of SpeedBeads™ carboxylate-modified magnetic particles (Merck, Darmstadt, Germany) and 135 µL of isopropanol were added to 100 µL of supernatant in a 96-well plate. The plate was placed on a magnetic rack for 15 min, supernatant was removed, and the beads were washed one time with 150 µL of isopropanol and then two times with 200 µL of 70% ethanol. The beads were air-dried and, after removal of the plate from the magnetic rack, resuspended in 50 µL RNAse free water for 3 min. The plate was placed back on the magnetic rack for 10 min to collect the eluate containing total RNA. Equine arteritis virus (EAV) RNA in AVL lysis buffer (Qiagen, Hilden, Germany) was spiked into the reagent as an internal control for extracellular RNA samples. The cellular reference gene PGK1 served as a control for intracellular RNA. Primers and probes for EAV and PGK1 and the normalization procedure were described previously [24]. Viral RNA was quantified by RT-qPCR using TaqMan Fast Virus 1-step master mix (Thermo Fisher Scientific, Waltham, MA, USA) as previously described [26]. Primers and probes for SARS-CoV-2 and SARS-CoV, as well as a standard curve, were used as described previously [26,27], and for MERS-CoV, with final primer concentrations of 450 nM each and probe concentrations of 200 nM. Angptl4 was quantified using the ANGPTL4 TaqMan^®^ Gene Expression Assay FAM-MGB (Thermo Fisher Scientific, Catalog No. 4331182, ID Hs01101123_g1).

## Data Availability

Data are contained within the article.

## References

[1] Bösmüller H., Matter M., Fend F., Tzankov A. (2021). The pulmonary pathology of COVID-19. Virchows Arch. Int. J. Pathol..

[2] Wang P., Luo R., Zhang M., Wang Y., Song T., Tao T., Li Z., Jin L., Zheng H., Chen W. (2020). A cross-talk between epithelium and endothelium mediates human alveolar–capillary injury during SARS-CoV-2 infection. Cell Death Dis..

[3] Bordoni V., Mariotti D., Matusali G., Colavita F., Cimini E., Ippolito G., Agrati C. (2022). SARS-CoV-2 Infection of Airway Epithelium Triggers Pulmonary Endothelial Cell Activation and Senescence Associated with Type I IFN Production. Cells.

[4] Solimando A.G., Marziliano D., Ribatti D. (2022). SARS-CoV-2 and Endothelial Cells: Vascular Changes, Intussusceptive Microvascular Growth and Novel Therapeutic Windows. Biomedicines.

[5] Ackermann M., Verleden S.E., Kuehnel M., Haverich A., Welte T., Laenger F., Vanstapel A., Werlein C., Stark H., Tzankov A. (2020). Pulmonary Vascular Endothelialitis, Thrombosis, and Angiogenesis in COVID-19. N. Engl. J. Med..

[6] Hassan M., Selimovic D., El-Khattouti A., Soell M., Ghozlan H., Haikel Y., Abdelkader O., Megahed M. (2014). Hepatitis C virus-mediated angiogenesis: Molecular mechanisms and therapeutic strategies. World J. Gastroenterol..

[7] Caposio P., Orloff S.L., Streblow D.N. (2011). The role of cytomegalovirus in angiogenesis. Virus Res..

[8] Bhatraju P.K., Morrell E.D., Stanaway I.B., Sathe N.A., Srivastava A., Postelnicu R., Green R., Andrews A., Gonzalez M., Kratochvil C.J. (2023). Angiopoietin-Like4 Is a Novel Marker of COVID-19 Severity. Crit. Care Explor..

[9] Sasaki M., North P.E., Elsey J., Bubley J., Rao S., Jung Y., Wu S., Zou M.-H., Pollack B.P., Kumar J. (2019). Propranolol exhibits activity against hemangiomas independent of beta blockade. NPJ Precis. Oncol..

[10] Zhang L., Mai H.M., Zheng J., Zheng J.W., Wang Y.A., Qin Z.P., Li K.L. (2014). Propranolol inhibits angiogenesis via down-regulating the expression of vascular endothelial growth factor in hemangioma derived stem cell. Int. J. Clin. Exp. Pathol..

[11] Varone F., Sgalla G., Iovene B., Bruni T., Richeldi L. (2018). Nintedanib for the treatment of idiopathic pulmonary fibrosis. Expert Opin. Pharmacother..

[12] Pang J., Xu F., Aondio G., Li Y., Fumagalli A., Lu M., Valmadre G., Wei J., Bian Y., Canesi M. (2021). Efficacy and tolerability of bevacizumab in patients with severe COVID-19. Nat. Commun..

[13] Zhang M., Wang P., Luo R., Wang Y., Li Z., Guo Y., Yao Y., Li M., Tao T., Chen W. (2020). Biomimetic Human Disease Model of SARS-CoV-2 Induced Lung Injury and Immune Responses on Organ Chip System. Adv. Sci..

[14] Li L., Chong H.C., Ng S.Y., Kwok K.W., Teo Z., Tan E.H.P., Choo C.C., Seet J.E., Choi H.W., Buist M.L. (2015). Angiopoietin-like 4 Increases Pulmonary Tissue Leakiness and Damage during Influenza Pneumonia. Cell Rep..

[15] Peuschel K.E. (2011). Some clinical evidence of the hypothesis of an indirect antiviral effect of propranolol through immunoactivation. Med. Hypotheses.

[16] Fang H., Wang Y., Liu L., Cheng K., Li P., Tan Y., Hao X., Mei M., Xu X., Yao Y. (2022). A Host-Harbored Metabolic Susceptibility of Coronavirus Enables Broad-Spectrum Targeting. bioRxiv.

[17] Stiles J., Amaya C., Pham R., Rowntree R.K., Lacaze M., Mulne A., Bischoff J., Kokta V., Boucheron L.E., Mitchell D.C. (2012). Propranolol treatment of infantile hemangioma endothelial cells: A molecular analysis. Exp. Ther. Med..

[18] Hu Q., Liao P., Li W., Hu J., Chen C., Zhang Y., Wang Y., Chen L., Song K., Liu J. (2021). Clinical Use of Propranolol Reduces Biomarkers of Proliferation in Gastric Cancer. Front. Oncol..

[19] Zhou C., Chen X., Zeng W., Peng C., Huang G., Li X., Ouyang Z., Luo Y., Xu X., Xu B. (2016). Propranolol induced G0/G1/S phase arrest and apoptosis in melanoma cells via AKT/MAPK pathway. Oncotarget.

[20] Klann K., Bojkova D., Tascher G., Ciesek S., Münch C., Cinatl J. (2020). Growth factor receptor signaling inhibition prevents SARS-CoV-2 replication. Mol. Cell.

[21] Salgado-Benvindo C., Leijs A.A., Thaler M., Tas A., Arbiser J.L., Snijder E.J., van Hemert M.J. (2022). Honokiol inhibits SARS-CoV-2 replication in cell culture. bioRxiv.

[22] Wang Y., Thaler M., Ninaber D.K., van der Does A.M., Ogando N.S., Beckert H., Taube C., Salgado-Benvindo C., Snijder E.J., Bredenbeek P.J. (2021). Impact of human airway epithelial cellular composition on SARS-CoV-2 infection biology. bioRxiv.

[23] Drosten C., Günther S., Preiser W., Van Der Werf S., Brodt H.-R., Becker S., Rabenau H., Panning M., Kolesnikova L., Fouchier R.A. (2003). Identification of a novel coronavirus in patients with severe acute respiratory syndrome. N. Engl. J. Med..

[24] Kovacikova K., Morren B.M., Tas A., Albulescu I.C., van Rijswijk R., Jarhad D.B., Shin Y.S., Jang M.H., Kim G., Lee H.W. (2020). 6′-β-Fluoro-Homoaristeromycin and 6’-Fluoro-Homoneplanocin A Are Potent Inhibitors of Chikungunya Virus Replication through Their Direct Effect on Viral Nonstructural Protein 1. Antimicrob. Agents Chemother..

[25] Ogando N.S., Dalebout T.J., Zevenhoven-Dobbe J.C., Limpens R., van der Meer Y., Caly L., Druce J., de Vries J.J.C., Kikkert M., Bárcena M. (2020). SARS-coronavirus-2 replication in Vero E6 cells: Replication kinetics, rapid adaptation and cytopathology. J. Gen. Virol..

[26] Salgado-Benvindo C., Thaler M., Tas A., Ogando N.S., Bredenbeek P.J., Ninaber D.K., Wang Y., Hiemstra P.S., Snijder E.J., van Hemert M.J. (2020). Suramin Inhibits SARS-CoV-2 Infection in Cell Culture by Interfering with Early Steps of the Replication Cycle. Antimicrob. Agents Chemother..

[27] Corman V.M., Landt O., Kaiser M., Molenkamp R., Meijer A., Chu D.K., Bleicker T., Brunink S., Schneider J., Schmidt M.L. (2020). Detection of 2019 novel coronavirus (2019-nCoV) by real-time RT-PCR. Euro Surveill.

